# Impact of the changes in chronic myeloid leukemia classification proposed by the 2022 World Health Organization: a single-center Brazilian study

**DOI:** 10.1016/j.htct.2026.106470

**Published:** 2026-05-16

**Authors:** Estela Gasparotto Emiliano, Gustavo Emmanuel Alves Abrantes Santos, Eliana Cristina Martins Miranda, Gislaine Borba Oliveira Duarte, Guilherme Brasil Duffles Amarante, Carmino Antônio De Souza, Katia Borgia Barbosa Pagnano

**Affiliations:** Universidade Estadual de Campinas (UNICAMP), Campinas, SP, Brazil

**Keywords:** Chronic myeloid leukemia, Imatinib, Survival, Accelerated phase, 2022 WHO classification

## Abstract

**Introduction:**

The latest edition of the 2022 World Health Organization (WHO) classification for chronic myeloid leukemia omitted the definition of accelerated phase.

**Methods:**

This study evaluated the impact of using the updated classification in chronic myeloid leukemia patients treated upfront with imatinib by analyzing the outcomes of 139 newly diagnosed patients, previously classified according to the 2017 WHO criteria.

**Results:**

Using the updated WHO Classification, eight (6%) cases previously characterized as accelerated phase were classified as chronic phase.

Overall survival (OS) was comparable under both the 2017 and 2022 WHO classifications. For patients in the chronic phase, OS was 86% at 60 months and 70% at 96 months (compared to 85% and 69% respectively under 2022 criteria). Conversely, outcomes were significantly poorer for advanced stages, with 0% survival for the accelerated phase and 25% for blast crisis (p-value <0.0001). Progression-free survival was 85% and 69% for the chronic phase and 25% for blast crisis (p-value <0.0001) by the 2017 WHO classification and 83% and 68% for chronic phase and 25% for blast crisis (p-value <0.0001) according to the 2022 WHO classification. Independent predictors of overall survival included basophil count, 2022 WHO classification, and age at tyrosine kinase inhibitor therapy initiation. For progression-free survival, the 2022 WHO classification (specifically blast crisis) and age at diagnosis were identified as significant factors.

**Conclusions:**

Age, elevated basophil count, and advanced disease at diagnosis remain associated with worse outcomes. The omission of the accelerated phase did not significantly affect the prognosis in this cohort. However, it may affect the treatment of individual patients in Brazil, since higher doses of tyrosine kinase inhibitors are indicated for those who meet the criteria for the previous accelerated phase definition.

## Introduction

Chronic myeloid leukemia (CML) is a myeloproliferative neoplasm that accounts for approximately 15–20% of adult leukemias [1, 2, 3, 4, 5]. CML is characterized by the reciprocal translocation t(9;22)(q34;q11), resulting in the fusion gene *BCR::ABL1,* which encodes a tyrosine kinase protein, the primary therapeutic target for tyrosine kinase inhibitors (TKIs) [2, 3, 4, 5, 6]. Classically, CML has been described as a triphasic disease: chronic phase (CP), accelerated phase (AP) and blast crisis (BC) [7]. CP is the initial stage, in which the percentage of blasts in the peripheral blood (PB) or bone marrow (BM) is <10%. Patients who do not respond to treatment with TKIs progress to the advanced stages of the disease (AP and BC), which morphologically resemble acute leukemia [2,3,5,7]. Currently, the 10-year overall survival (OS) rate for CML is estimated to be 80%−90% [8].

In the 2017 World Health Organization (WHO) Classification, AP was defined as persistent neutrophilia, splenomegaly, and/or thrombocytosis; persistent thrombocytopenia unrelated to therapy; 20% or more basophils in PB; 10–19% blasts in PB and/or BM; additional clonal cytogenetic abnormalities (ACAs) of Philadelphia positive (Ph+) cells at diagnosis in the major route (according to Ph+ cells, trisomy of chromosome 8, isochromosome 17q, trisomy 19); complex karyotype and 3q26.2 abnormalities; or any new clonal abnormality that occurs during therapy. Progression to BC is defined by ≥20% blasts in the BM or PB, or the infiltrative proliferation of extramedullary blasts. However, the detection of any lymphoblasts in the PB or BM, even at levels below 10%, indicates lymphoid blast crisis (Table 1) [7].

**Table 1:** 2017-WHO, 2022-ICC, 2020-ELN and 2022-WHO classification for Chronic Myeloid Leukemia (CML).

	2017-WHO [7]	2022-ICC [9]	2020-ELN [10, 11, 12]	2022-WHO [13]
**Accelerated phase**	PB or BM blasts 10%−19%		PB or BM blasts 15%−29% PB blasts + promyelocytes ≥30%, with blasts <30%	Not recognized
	PB basophils ≥20%			
		Not included	Persistent thrombocytopenia (≤100 ^x^ 10^9^/L) unrelated to therapy	
	Persistent or increasing splenomegaly (unresponsive to therapy)	Not included	Not included	
	ACA in pH^+^ cells at diagnosis, including major route, complex karyotype, or 3q26.2 abnormalities, at diagnosis Any new clonal chromosomal abnormality in pH+ cells that occurs during therapy	ACA in Ph+ cells at diagnosis or the acquisition of major route ACAs on treatment	Clonal chromosome abnormalities in Ph+ cells (CCA/Ph+), major route, on treatment	
	Provisional: - Failure to achieve CHR to first TKI - Any indication of resistance to two sequential TKIs - Occurrence of >2 mutations of BCR::ABL1 during TKI treatment	Not included	Raises concern: - ACAs in Ph+ cells - Resistance to two TKIs - Detection of a *BCR::ABL1* kinase domain mutation	
**Blast crisis**	PB or BM blasts ≥20%		PB or BM blasts ≥20%	PB or BM blasts ≥20%
	Extramedullary blast proliferation, apart from spleen	Myeloid sarcoma	Extramedullary blast proliferation, apart from spleen	Myeloid sarcoma
		Presence of morphologically apparent lymphoblasts (>5%) warrants consideration of lymphoid BP-CML	Not included	Presence of increased lymphoblasts in PB or BM

WHO: World Health Organization; ICC: International Consensus Classification; ELN: European LeukemiaNet; ACA: additional clonal cytogenetic abnormalities; BM: bone marrow; CHR: complete hematologic remission; PB: peripheral blood; WBC: white blood cells; TKI: Tyrosine kinase inhibitor; Ph+: Philadelphia positive.

However, the new WHO classification of 2022 omitted the AP definition, emphasizing mainly the factors associated with high risk that mark the transition from CP to BC, such as the accumulation of mutations, ACAs, and resistance to TKIs. The exclusion of AP was also justified by the decrease in the number of patients who progress to advanced phases after TKI therapy, which has increased the overall survival (OS) rate thereby improving the prognosis of AP patients [13].

On the other hand, the 2022 International Consensus Classification (ICC) of Myeloid Neoplasms and Acute Leukemias maintained the triphasic disease definition. The identification of ACAs marks the transition from CP to AP, characterized mainly by 10–19% blasts in the bone marrow or peripheral blood, in addition to 20% or more basophils in peripheral blood [9].

The differences between the definitions of the two classifications may influence treatment decisions and prognostic analyses, as most studies that approved the current TKIs had used the definition of AP to include patients in clinical trials [14].

This study aimed to evaluate the impact of the 2022 WHO classification on the categorization and prognosis of CML patients treated with first-line imatinib.

## Methods

This was a single-center, observational, retrospective study. This study analyzed electronic medical records of newly diagnosed CML patients at treated at Centro de Hematologia e Hemoterapia, Universidade Estadual de Campinas, a Brazilian university institution (from January 2015 to December 2023). Participants received first-line imatinib (400–600 mg/day) and underwent regular clinical follow-ups. Clinical outcomes were compared across the 2017 WHO, 2022-ICC, and 2022 WHO classifications. Cases with insufficient clinical and laboratory data to calculate the disease stage before starting treatment were excluded. This study was approved by the local Institutional Review Board.

OS was calculated from the start of imatinib administration until the date of death or last follow-up. Progression-free survival (PFS) was calculated from the start of imatinib therapy until transformation to AP or BC or death. Survival was analyzed using the Kaplan-Meier method, and differences between curves were assessed using the log-rank test. The cut-off date for this analysis was May 2024. Patients were stratified using the European Treatment and Outcome Study (EUTOS) long-term survival score (ELTS) [15] and the Sokal score [16]. Responses were analyzed according to the definitions of the European LeukemiaNet (ELN) recommendations 2006–2020 [10, 11, 12].

## Results

**Figure 1:**
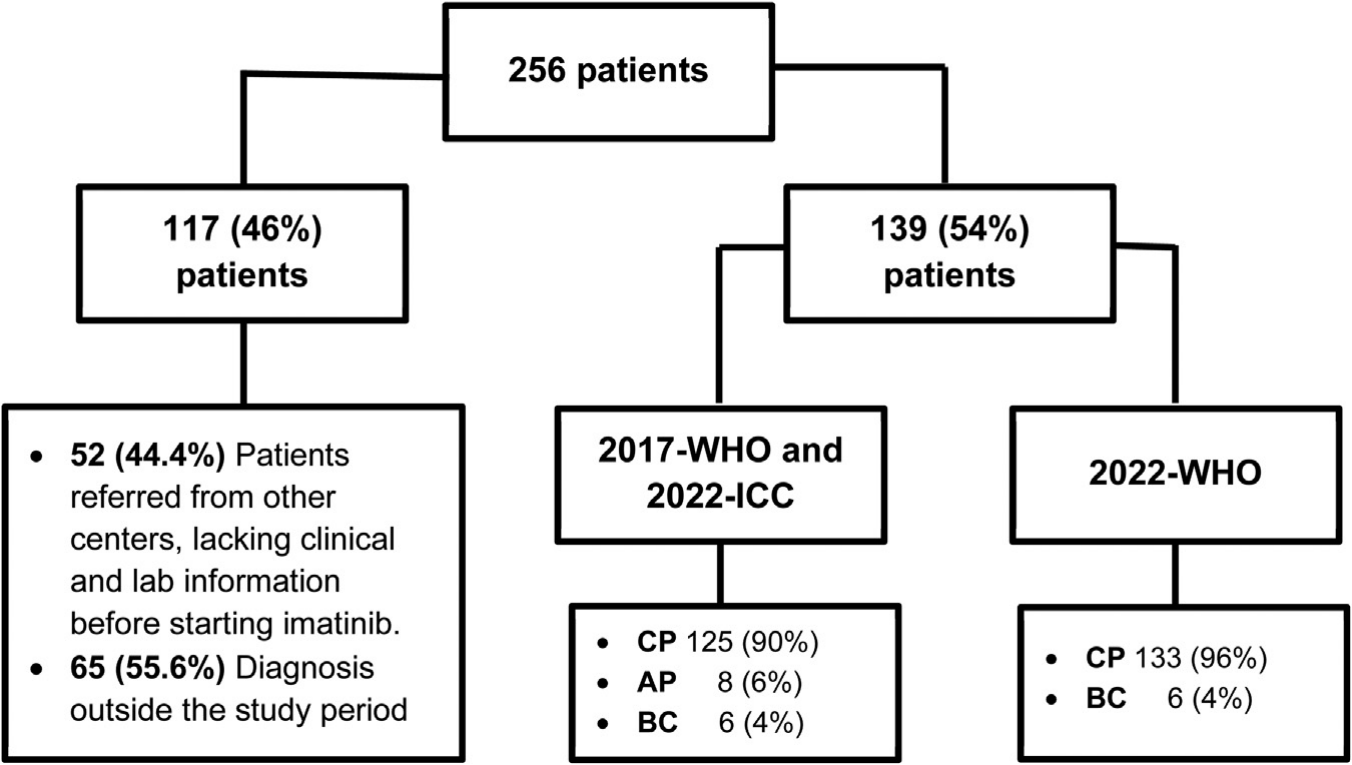
Flowchart of patients with chronic myeloid leukemia treated with Imatinib (n = 256).

A total of 256 electronic medical records of patients followed at the center were analyzed (Figure 1), of which 117 (46%) were excluded for the following reasons: diagnosis outside the study period and lack of clinical information before starting imatinib therapy (patients referred from other centers). The clinical and laboratory characteristics of the 139 (54%) patients evaluated in this study are described in Table 2.

**Table 2:** Clinical and laboratorial data on chronic myeloid leukemia patients at diagnosis (n = 139).

Variable	
Age (years) - median (range)	53 (17–83)
Sex, female – n (%)	71 (51)
Spleen size (cm from BCM) - median (range)	0 (0–30)
Laboratory data - median (range)	
White blood cell count (x 10^9^/L)	117.2 (5.4–588)
Basophils (%)	3.2 (0–18)
Blasts in peripheral blood (%)	2 (0–60)
Platelets (x 10^9^/L)	387 (71–3862
Hemoglobin g/dL	11.4 (3.5–16.6)
Eosinophils (%)	2 (0–6)
Bone marrow blasts (%)	1 (0–63)
Bone marrow basophils (%)	1.5 (0–15)
Sokal score – n (%)	
Low	37 (26.6)
Intermediate	59 (42.4)
High	39 (28.1)
Not available	4 (2.9)
ELTS – n (%)	
Low	55 (39.6)
Intermediate	47 (33.8)
High	33 (23.7)
Not available	4 (2.9)

BCM: Below costal margin; ELTS: European long-term survival score.

Additional chromosomal abnormalities (ACA) were found in 25/139 (18%) patients: seven (28%) were major route alterations, specifically of trisomy of chromosomes 8 and 19 and isochromosome [i(17q)], four (16%) other high-risk alterations (trisomy 21, 3q26.2, monosomy 7/7q−, 11q23, complex karyotypes) and 14 (56%) low-risk alterations (any other).

BCR::ABL1 transcripts were identified in 133 (95.5%) of the 139 cases: b3a2 in 84 (63.2%) patients, b2a2 in 46 (34%), both in two (1.4%), e1a2 in one (0.7%), and p230 in one (0.7%) case. Two patients died before starting imatinib treatment and 137 started imatinib in a median time of ten days.

According to the 2017 WHO and 2022-ICC classifications, 125 (90%) were in the CP, eight (6%) in the AP, and six (4%) in the BC phase; and according to 2022 WHO, 133 (96%) were in the CP and six (4%) in the BC phase (Figure 1). There was a significant change in the 2022 WHO classification compared to the 2017 version (p-value <0.0001%). Eight of 139 (6%) patients were reclassified from AP to CP.

### Treatment response

In the entire group, the response to first-line imatinib treatment was as follows: complete hematologic response (CHR) was achieved by 135 (97%) patients; complete cytogenetic response (CCyR) by 83 (60%) and major molecular response (MMR) by 65 (46.5%). Fourteen (10%) patients lost CHR, seven (8.5%) lost CCyR and 15 (23%) lost MMR. Overall, 11 (8%) patients progressed to the advanced phases, four to AP and seven to BC. Eleven (8%) required hematopoietic stem cell transplantation (HSCT). There were 22 (16%) deaths, 15 (68%) of which were CML related.

Of the eight patients in AP who were reclassified as CP, five switched to a second-generation TKI because of imatinib failure, and two of them required allogeneic HSCT; one of whom died from the procedure. One patient progressed to BC, one patient was lost to follow-up, and four are currently under treatment (Table 3).

**Table 3:** Summary of clinical characteristics of chronic myeloid leukemia (CML) patients who were reclassified from accelerated phase (AP) (2017-WHO/ 2022-ICC) to chronic phase (CP) by 2022-WHO.

Patient #	Sex	Age at diagnosis, (years)	Blasts in PB at diagnosis (%)	BM blasts at diagnosis (%)	BM basophils at diagnosis (%)	Progression to advanced phases	Response to first-line treatment with imatinib	Second-generation TKI during follow-up	Disease status in the last follow-up	Treatment in the last Follow-up (dose mg)	Situation in the last Follow-up
1	F	50	12	10.5	4.5	No	CHR	Yes	CHR	Post HSCT	Deceased
2	M	36	13	2.5	1.5	No	CHR (lost to follow-up)	Yes	MMR	Nilotinib (800)	Alive - Transferred to other hospital
3	F	69	11	8.2	0.8	Yes	CHR	Yes	BC	Dasatinib (100)	Deceased
4	F	57	13	0	5	No	CHR CCyR MMR	No	MMR	Imatinib (600)	Alive - on treatment
5	F	58	11	1.5	1	No	CHR	Yes	DMR	Dasatinib (100)	Alive - on treatment
6	M	30	12	12	5	No	CHR	Yes	PMR	Post HSCT	Alive - on treatment
7	F	69	19	15.8	1.8	No	CHR CCyR MMR	No	DMR	Imatinib (600)	Alive - Lost follow-up
8	M	58	4	12	3.5	No	CHR CCyR MMR	No	MMR	Imatinib (600)	Alive - on treatment

PB: Peripheral blood; BM: Bone marrow; TKI: tyrosine kinase inhibitor; F: female; M: male; allo-HSCT: allogeneic hematopoietic stem cell transplantation; CHR: Complete hematologic response; CCyR: Complete cytogenetic response; MMR: Major molecular response; DMR: Deep molecular response.

**Figure 2:**
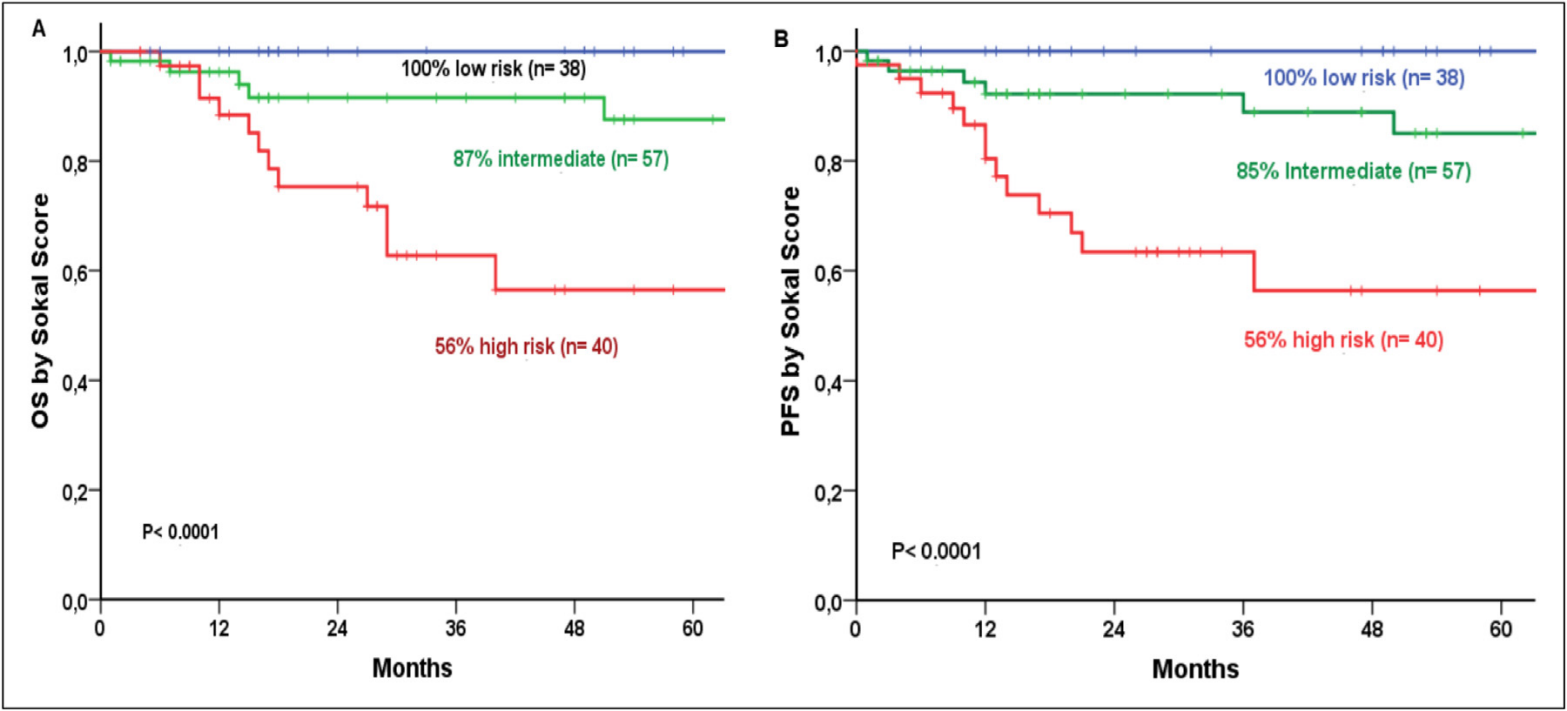
Overall Survival (2A) and Progression-Free Survival (2B) by Sokal score.

**Figure 3:**
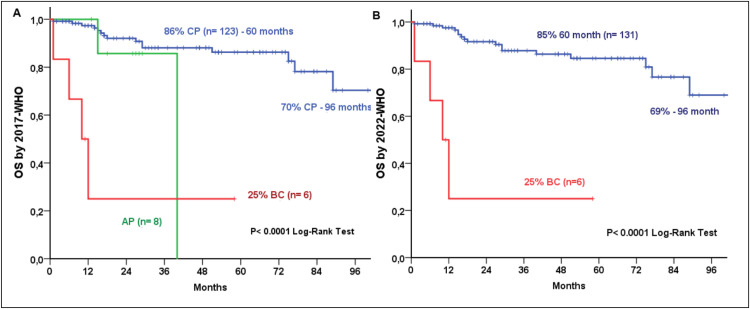
Overall Survival by 2017 WHO (A) and 2022 WHO (B) classifications.

**Figure 4:**
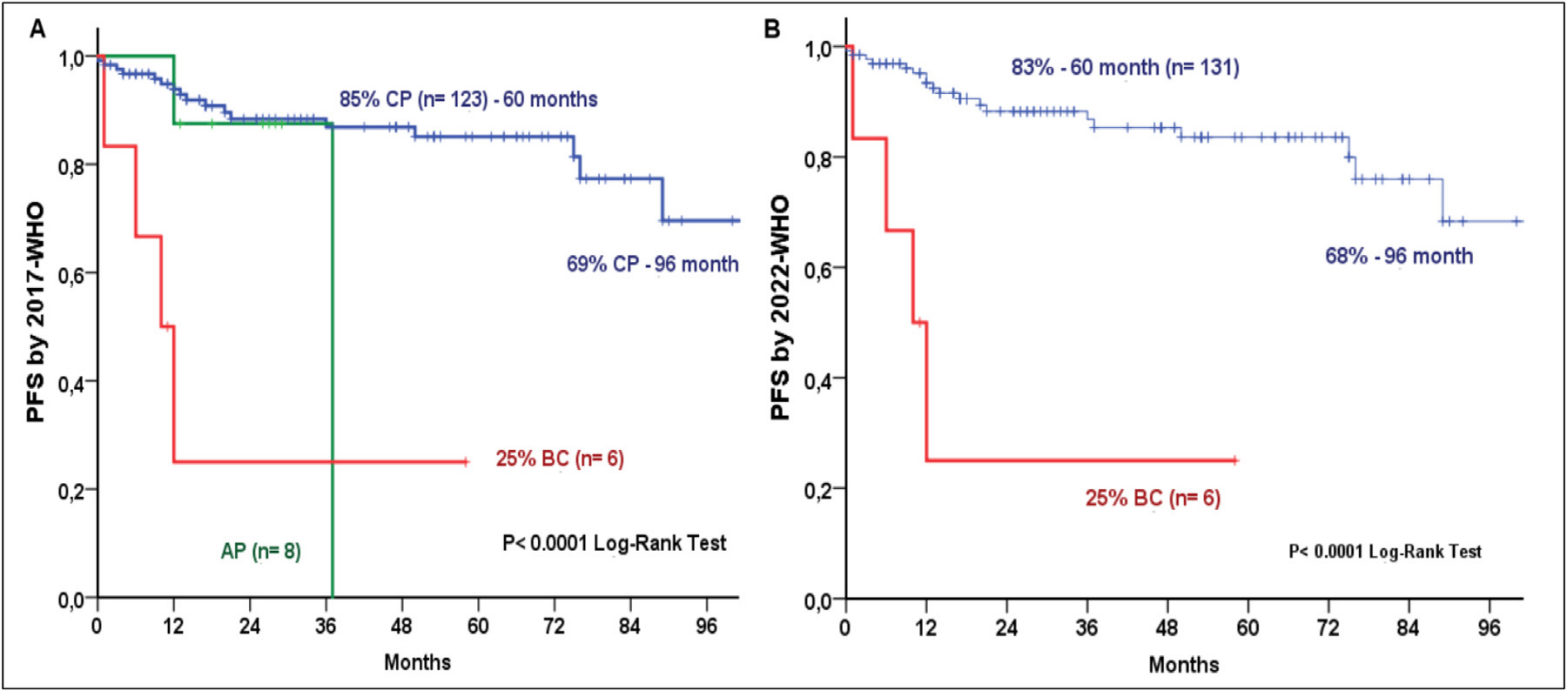
Progression-Free Survival by 2017 WHO (A) and 2022 WHO (B) classifications.

Based on Sokal risk scores, OS was 100%, 87%, and 56% for low, intermediate, and high-risk groups, respectively, while corresponding PFS rates were 100%, 85%, and 56% (both p-value <0.0001; Figure 2). Survival outcomes remained consistent across classifications: under 2017 WHO/2022 ICC criteria, CP OS was 86% at 60 months and 70% at 96 months, compared to 0% for AP and 25% for BC (p-value <0.0001; Figure 3). Under 2022 WHO criteria, CP OS was 85% and 69% at 60 and 96 months, respectively and 25% for BC (p-value <0.0001; Figure 3). Similarly, 2017 WHO/2022 ICC PFS was 85% (60 months) and 69% (96 months) for CP, 0% for AP, and 25% for BC (p-value <0.0001; Figure 4), while 2022 WHO PFS was 83% (60 months) and 68% (96 months) for CP and 25% for BC (p-value <0.0001; Figure 4). Multivariate analysis identified basophil count (p-value = 0.013), 2022 WHO phase, and age at TKI initiation (p-value = 0.019) as independent predictors for OS. For PFS, 2022 WHO specifically BC (p-value <0.0001) and age at diagnosis were the sole independent factors (p-value = 0.010)

## Discussion

The omission of the AP classification did not significantly affect OS or PFS rates in CML patients treated with first-line imatinib in the present cohort. Age, higher basophil count, and advanced disease at diagnosis were independent factors of worse outcomes. This finding corroborates the literature, which argues that it is essential to develop predictive tools to quickly identify patients at higher risk of CML-induced progression and death, warranting closer molecular monitoring and treatment intensification, such as frontline second generation TKIs, early switch to a more potent TKI, eligibility for allogeneic HSCT, or new agents [17].

The rationale for the change to the 2022 WHO classification was the improvement of CML survival in the last decades after TKI therapy introduction. AP was omitted in the updated classification, but high-risk features associated with CP progression and resistance to TKI were stressed [13].

A possible alternative to complete exclusion could be to simplify the definition of AP, as defined by the 2022-ICC. Variables that were considered less-specific definers of AP (platelet count and persistent splenomegaly unresponsive to therapy) were omitted due to more robust evidence of the association of other parameters with a higher risk of progression to worse outcomes [14]. There is a need for further discussion in this respect, as it remains a heterogeneous disease with marked variability in individual prognosis [18].

In a real-world multicenter study [19] using the Czech 'INFINITY' registry, patients diagnosed with AP (by 2020-ELN, 2022-ICC, and 2017 WHO criteria) had a significantly worse prognosis than those in the CP group, even those classified as high-risk by the ELTS. These findings support the recognition of AP as a distinct clinical entity at diagnosis. In the current cohort of AP patients, only three of eight are still on imatinib, and five have switched to a second-generation TKI due to resistance.

On the other hand, Yang et al. [20] analyzed 2122 consecutive CML patients diagnosed between 2006 and 2023. Using the 2022 ICC, they found that response milestones, OS, and PFS, were consistent with results obtained using the 2020-ELN criteria. AP patients with increased basophil counts had similar transformation-free survival (TFS) and OS compared with CP subjects classified as intermediate-risk by the ELTS, whereas AP patients with increased blasts had worse TFS but similar OS compared with those with CP and high-risk ELTS. Similar findings were observed using the 2022-ICC. The authors supported omitting AP but suggested updating the ELTS risk classification to include a very high-risk cohort with increased blasts and/or decreased platelets.

Kantarjian et al. reviewed the outcomes of both *de novo* CML-AP and ‘transformed’ CML-AP (evolving from CML-CP) treated in the era before TKI therapy (before 2000) and in the TKI therapy era (after 2000). There was an improvement in survival from 30%−66% in *de novo* CML patients with AP, but less progress in transformed AP. The authors noted that many clinical trials used the AP definition for patient stratification, and omitting AP may impair patient recruitment and coverage of treatment costs [18].

The omission of AP presents not only theoretical but also practical implications. It may also influence ongoing scientific research with TKI-based regimens or new therapies, as well as community practice in CML, including considering HSCT, since pathologists who follow the 2022 WHO criteria will not report progression to AP. These patients cannot be included in such protocols [18].

The potential challenges that the omission of AP for individual patients in Brazil should not be underestimated, as, in the public healthcare system in Brazil, only imatinib is available as the first-line treatment for CML and higher doses of imatinib are only reimbursed using the previous definition of AP and not for high-risk CP patients according to ELTS.

## Conclusions

Age, higher basophil counts, and advanced disease at diagnosis remain relevant factors associated with worse treatment outcomes. Omitting the AP from the 2022 WHO classification did not significantly impact OS or PFS in this cohort of CML patients treated with first-line imatinib. However, its exclusion may influence individual clinical management, which has historically relied on the AP definition for treatment decisions.

## Funding

This study was supported by the National Council for Scientific and Technological Development (CNPq) through the Institutional Scientific Initiation Scholarship Program (PIBIC) Quota 2023/2024.

## Conflicts of interest

None
